# When the Heart's Blueprint Goes Awry: Exploring Congenital Absence of the Left Circumflex Artery in a Case of Chronic Dyspnea

**DOI:** 10.1002/ccr3.71714

**Published:** 2025-12-19

**Authors:** Rohit Pandit, Ahmad Abu‐Haniyeh, Anil Nepali, Nishchal Regmi, Heena Maharjan, Ahmad El‐Moussa

**Affiliations:** ^1^ TidalHealth Peninsula Regional, Internal Medicine Salisbury Maryland USA; ^2^ Kathmandu University School of Medical Sciences Dhulikhel Nepal; ^3^ KIST Medical College Lalitpur Nepal

**Keywords:** case report, chronic shortness of breath, congenital absence of left circumflex artery, coronary artery anomalies, hyperdominant right coronary artery

## Abstract

Congenital absence of the left circumflex artery (CALCx) is a rare coronary anomaly that is often asymptomatic but sometimes presents with nonspecific symptoms that may be mistaken for obstructive coronary artery disease. We present the case of a 59‐year‐old woman with a long‐standing history of exertional dyspnea and recent onset of palpitations, orthopnea, and chest heaviness. Her vital signs on presentation were blood pressure 132/78 mmHg, heart rate 86 bpm, respiratory rate 16/min, temperature 36.6°C, and oxygen saturation 96% on room air. Her cardiovascular risk factors included hypertension, hyperlipidemia, obesity, and obstructive sleep apnea. Initial workup, including ECG and stress testing, revealed nonspecific changes and ventricular ectopy. Coronary angiography and CT angiography confirmed CALCx, with a hyperdominant right coronary artery supplying the LCx territory and mild atherosclerotic changes. Her coronary calcium score of 95.1 represented a mild‐to‐moderate atherosclerotic burden relevant to long‐term risk stratification. The patient's symptoms were attributed to a supply–demand mismatch due to the anatomical variant, rather than obstructive coronary disease. She was managed conservatively with medical therapy and risk factor modification, leading to gradual symptomatic improvement. This case is unique because it presented with chronic dyspnea without evidence of ischemia, unlike prior reports describing myocardial infarction, syncope, or emotional stress–related chest pain. This case highlights the importance of considering rare congenital anomalies like CALCx in the differential diagnosis of chronic dyspnea and nonspecific cardiac symptoms. Accurate diagnosis via coronary CTA or invasive angiography is essential to avoid misdiagnosis and guide management. Individualized, conservative treatment may be appropriate in the absence of hemodynamically significant lesions.

## Key Clinical Message

Congenital absence of the left circumflex artery can mimic atherosclerotic occlusion and is best identified with coronary CT angiography or invasive angiography. Correct recognition prevents misdiagnosis and guides individualized management, especially since no standardized diagnostic or treatment guidelines exist for this anomaly.

## 1 Introduction

Congenital absence of the left circumflex (CALCx) artery is an extremely rare cardiac defect, characterized by the absence of the left circumflex artery on angiogram, which is compensated by a hyperdominant right coronary artery to supply the posterolateral wall of the left ventricle. The overall frequency of CALCx is 0.003% [1].

In about 81% of patients, coronary artery anomalies are asymptomatic and benign, which do not lead to clinical complications and are discovered incidentally during angiography. In many cases, this anomaly may be misdiagnosed as atherosclerotic occlusion of the left circumflex artery, complicating both diagnosis and management. These anomalies sometimes present with clinical features such as exertional syncope, exercise‐induced arrhythmias, myocardial infarction, or cardiac arrest requiring surgical intervention [2]. The gold standard and widely available diagnostic tool for coronary anomalies is conventional coronary angiography (CCA). However, multidetector computed tomography (MDCT), the use of which has recently increased, allows for noninvasive visualization of coronary anatomy owing to the increased prevalence of coronary anomalies [1, 3].

This case report explores the clinical presentation, diagnostic challenges, and management of a 59‐year‐old woman with a long‐standing history of exertional dyspnea and recent onset of chest heaviness, palpitations, and orthopnea. Her cardiovascular risk factors and a comprehensive workup led to the discovery of CALCx, a condition that was initially misinterpreted as obstructive coronary artery disease.

## 2 Case Report

### 2.1 Clinical History and Examination

A 59‐year‐old woman with a past medical history of hypertension, hyperlipidemia, prediabetes, class III severe obesity, CPAP‐managed obstructive sleep apnea, and pernicious anemia with vitamin B12 insufficiency presented for evaluation of non‐radiating generalized chest heaviness with worsening shortness of breath, particularly when lying flat. She had been experiencing progressive exertional dyspnea for 3–4 years. Her symptoms were now associated with intermittent palpitations and a subjective sensation of missed beats, occasionally relieved with a cold shower. Additionally, she had persistent indigestion refractory to antacids.

Because indigestion and epigastric discomfort may represent anginal equivalents, and given her cardiovascular risk factors, prioritizing cardiac evaluation was appropriate and consistent with the 2021 AHA/ACC Chest Pain Guideline. After cardiac causes were excluded, her indigestion, which was long‐standing, non‐exertional, nonprogressive, and without alarm features, was consistent with functional dyspepsia; per the ACG/CAG Dyspepsia Guideline, urgent GI referral is not indicated in patients under 60 without alarm features.

She denied substernal chest pain, jaw discomfort, dizziness, diaphoresis, or edema. She had no personal or family history of cardiac disease. She was a never‐smoker and consumed alcohol rarely.

#### 2.1.1 Vital Signs

BP 132/78 mmHg, HR 86 bpm, RR 16/min, Temp 36.6°C, SpO_2_ 96% RA. Cardiac examination revealed normal S1 and S2, without murmurs.

### 2.2 Differential Diagnosis, Investigations and Treatment

Based on the history and examination, acute coronary syndrome, ANOCA, and congenital coronary anomalies were suspected.

**FIGURE 1:**
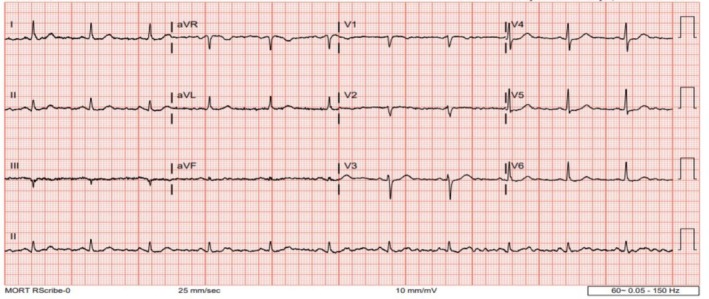
Twelve‐lead electrocardiogram (ECG) demonstrating sinus rhythm with nonspecific ST‐T wave abnormalities. Mild ST‐segment depression is noted in leads V4 and V5, with dynamic T‐wave flattening in leads V1 and V2.

ECG showed sinus rhythm with nonspecific ST and T wave abnormalities (Figure 1). Labs were normal, and chest radiography was unremarkable.

She underwent an exercise tolerance test: she walked 3 min and 29 s, achieving a max heart rate of 146 bpm. The test was stopped due to fatigue, dyspnea, and frequent ventricular ectopy, including PVCs, couplets, and a 3–4 beat run of ventricular tachycardia. ST depression of 1.0 mm was noted in inferolateral leads. A nuclear stress test revealed a medium‐sized anterior attenuation artifact due to breast tissue, preserved systolic function (60%), and no wall motion abnormalities.

She was started on metoprolol XL 25 mg, amlodipine 5 mg, rosuvastatin 20 mg, and aspirin 81 mg. Metoprolol was selected specifically for suppression of ventricular ectopy, rate control, and reduction of myocardial oxygen demand.

**FIGURE 2:**
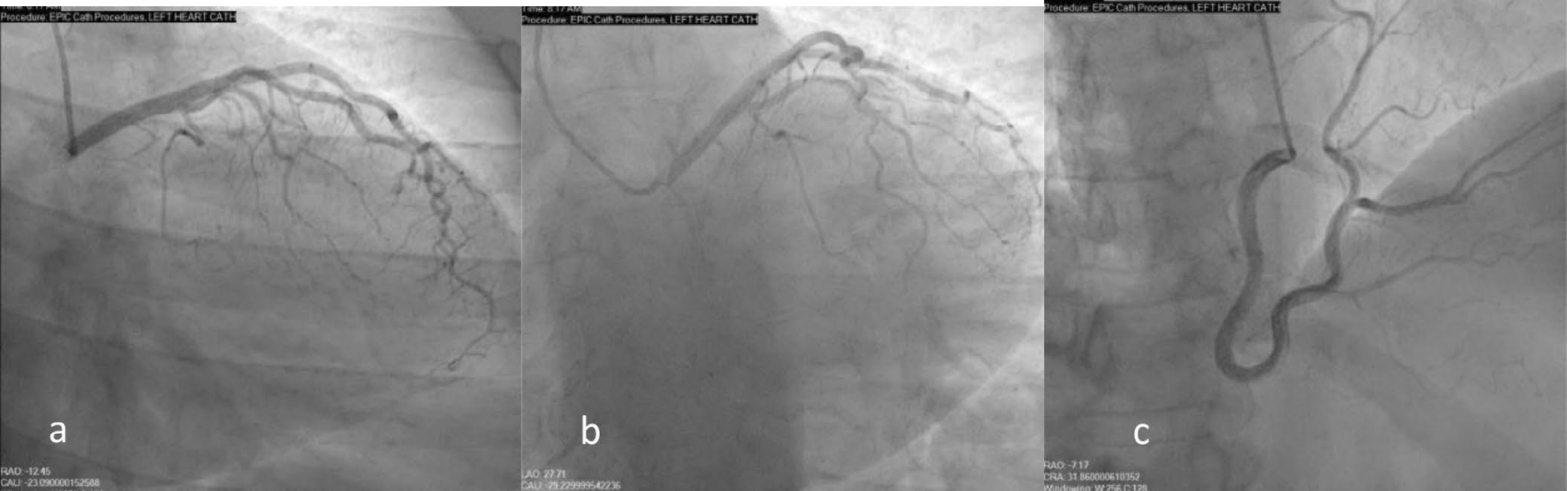
Coronary angiography. Right (a) and left (b) anterior oblique caudal view demonstrating absence of the LCx. (c) Right anterior oblique cranial view showing a dominant RCA supplying the territory typically perfused by the left LCx.

Coronary angiography revealed congenital absence of the LCx, a hyperdominant RCA, and no obstructive CAD (Figure 2). LVEDP was 9 mmHg, and EF was 60%–65%. Aortic root injection showed LAD as the sole branch of the left main.

**FIGURE 3:**
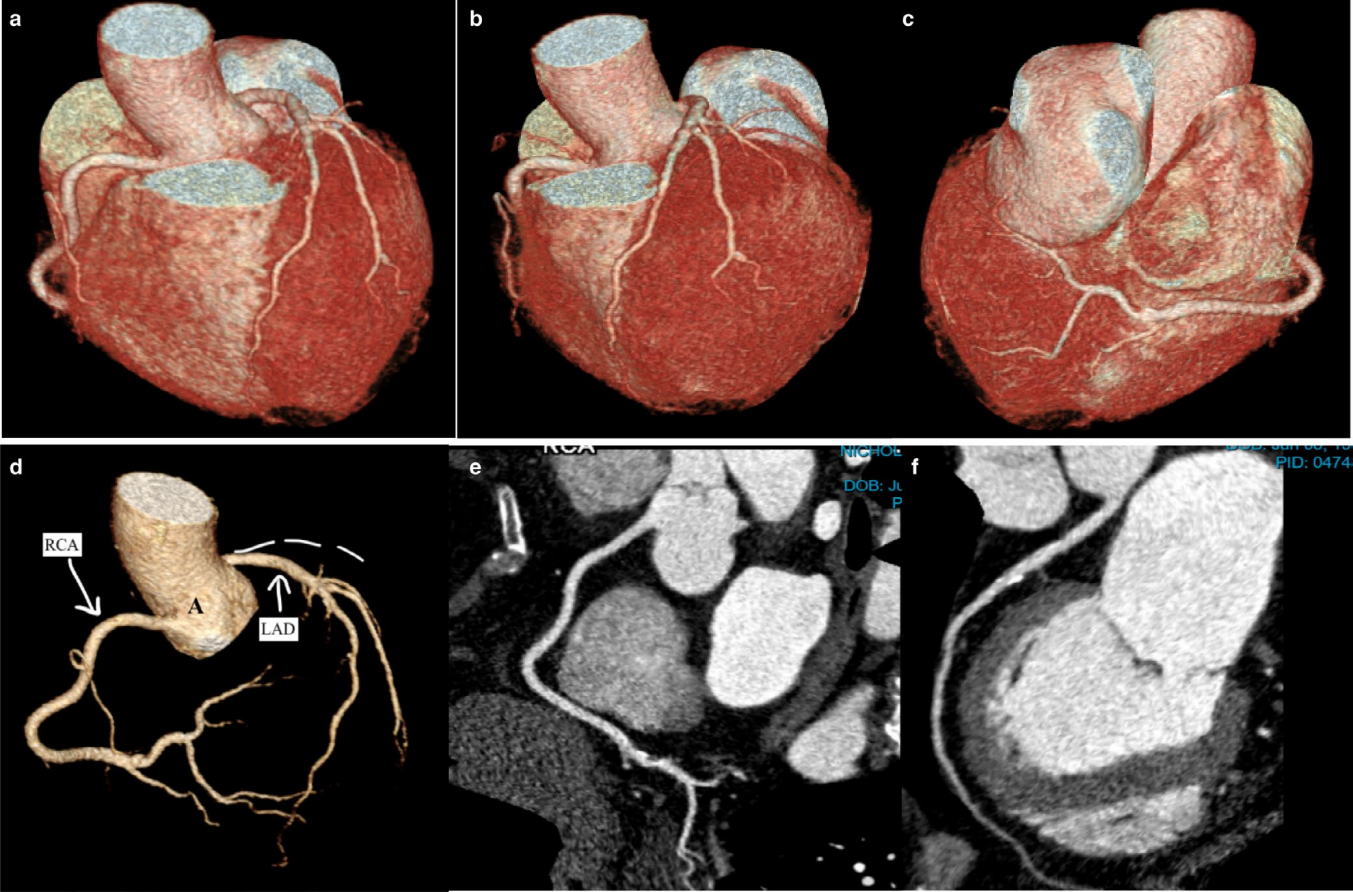
3D volume rendering coronary CT images (a–d): Panels (a–c) show the absence of the LCx, with its territory instead supplied by a hyperdominant RCA and diagonal branches of the LAD. The left main continues as the LAD, and the RCA is present with a large posterolateral branch crossing the heart's crux. Panel (d) displays the aortic root (A), LAD, hyperdominant and enlarged RCA, and absent LCx is not visible; the ordinary position of the LCx is marked as a dotted line, highlighting the single LAD and absence of LCx in the interventricular groove. Curved Planar Reformation (CPR) images Panels (e and f) depict: (e) the hyperdominant RCA crossing the crux and supplying the LCx territory, and (f) the LM coronary artery originating from the aorta and continuing as the LAD.

Computed tomography angiography (CTA) confirmed absence of the LCx with a hyperdominant RCA and revealed mild calcifications in the LAD and RCA, and mild–moderate stenosis of the second diagonal branch (Figure 3). The calcium score of 95.1 indicated a mild‐to‐moderate atherosclerotic burden relevant to long‐term cardiovascular risk.

### 2.3 Conclusion and Results

Diagnosis: congenital absence of LCx with hyperdominant RCA. ANOCA was ruled out. She was maintained on metoprolol, aspirin, amlodipine, and rosuvastatin with risk‐factor modification. Her dyspnea gradually improved over time. Routine cardiology follow‐up was arranged at one year.

## 3 Discussion

Coronary artery anomalies (CAA) are rare findings, with an incidence of 1.3%–2.06% on coronary angiography, which is an invasive diagnostic tool [2, 4]. Recently, after the development of ECG‐gated 64‐slice multidetector‐row computed tomography, a test that allows accurate and noninvasive detection of coronary artery anomalies, the incidence is 2.33% [5]. Coronary artery anomalies are the second most common cause of sudden cardiac death in young athletes [6]. Among those anomalies, the congenital absence of the left circumflex artery (CALCx) is extremely rare. In 0.67% of cases, the most frequent abnormality was the left circumflex artery's originating from the distal right coronary artery. CALCx is due to the failure of the development of the left circumflex artery from the atrioventricular groove [6, 7].

Several clinical presentations of CALCx, ranging from being asymptomatic to acute or chronic coronary syndromes, particularly angina‐like symptoms, might be confused with atherosclerotic occlusion of the left circumflex artery. The exact pathophysiology of angina‐like symptoms is unknown. However, the most likely reason might be a “supply–demand mismatch” or “steal phenomenon.” In this phenomenon, increased supply to the left circumflex artery results in transient ischemia in areas supplied by other coronary arteries [7]. The patient in our report presented with almost four years of shortness of breath with a new episode of chest heaviness and palpitations.

For the diagnosis of coronary artery anomalies, at resting heart rate, radiological information on cardiac anatomy is provided by coronary angiogram and cardiac CTA. Cardiac CTA is currently indicated in asymptomatic high‐risk patients, particularly with inconclusive stress tests. In distinguishing the ostial origin and proximal course of aberrant coronary arteries, multidetector computed tomography (MDCT) is superior to conventional angiography. The same is true in patients with myocardial bridging who present with chest pain or even myocardial infarction. In some patients, an exercise stress test or a radionuclide test of dobutamine stress is useful for functional assessment [8]. Positron emission tomography (PET) using Rubidium‐82 has better diagnostic accuracy and spatiotemporal resolution than single‐photon emission computed tomography myocardial perfusion imaging (SPECT), especially in obese patients [9]. When preliminary tests are inconclusive or when there is a strong clinical suspicion of ischemia that has not been clarified, advanced imaging such as stress MRI or PET is usually utilized [10, 11].

In this case, CTA was performed after coronary angiography not because angiography was insufficient, but because CTA provides superior anatomical delineation for confirming true congenital absence versus anomalous origin.

The patient's chronic dyspnea—with preserved EF, absence of ischemia on nuclear imaging, and lack of wall‐motion abnormalities—did not suggest functionally significant ischemia; therefore, additional MRI/PET testing was not pursued, consistent with guideline‐directed evaluation.

The clinical picture in our case did not indicate a high likelihood of significant ischemia, so additional testing with stress cardiac MRI or PET was not pursued, even though functional ischemia in the circumflex distribution was taken into consideration. The patient had preserved left ventricular systolic function, no exertional chest pain, and no obvious regional wall motion abnormalities or high‐risk characteristics detected by the nuclear stress test. The risk of flow‐limiting coronary stenosis is very low because neither coronary CT angiography nor invasive angiography showed any obstructive disease.

Table 1 highlights the diagnostic criteria for the absence of the left circumflex artery based on angiographic data or autopsy findings [1]. As there are no established guidelines, management of patients with coronary artery anomalies is controversial. In asymptomatic patients, no routine testing is required to rule out a coronary anomaly.

**TABLE 1:** Diagnostic criteria for the absence of the left circumflex artery, based on angiographic data or autopsy findings.

1. Absence of LCx origin: The LCx does not originate from either the left coronary sinus as a separate vessel or from the left main coronary artery (LMCA)
2. No anomalous origin: The LCx is not found arising anomalously from the right coronary sinus, the right coronary artery (RCA), the noncoronary sinus, or the pulmonary artery
3. Absence of collateral circulation: No collateral vessels compensate for the absent LCx from other coronary territories
4. Super‐dominant RCA: The RCA is notably enlarged (super‐dominant), supplying the posterolateral territory of the left ventricle through additional branches that traverse the posterior atrioventricular (AV) groove

In symptomatic patients with a high risk of sudden death, particularly male athletes, cardiac catheterization should be done even if the echocardiogram and treadmill stress test are negative [12]. In symptomatic young patients (< 35 years of age), revascularization is advised to prevent sudden death. In symptomatic patients older than 35 years of age, who are probably at insignificant risk of sudden death, management should be focused on treating symptoms. Management of each patient should be individualized as the magnitude of risk is unknown [8].

In our case, a coronary angiogram and coronary CT angiography were done for ECG findings and inconclusive nuclear stress tests. As coronary angiography is the gold standard and the patient was hemodynamically stable, it was done initially. Later, CT angiography was performed to conduct a detailed anatomical evaluation of the coronary arteries. With the finding of a congenital absence of the left circumflex artery (LCx), a normal ejection fraction, and normal left ventricular end‐diastolic pressure, conservative management was pursued. ANOCA was another possibility as per the patient's metabolic syndrome, which includes obesity, hypertension, dyslipidemia, and glucose intolerance, as well as her symptoms and abnormal stress test results. However, neither a noninvasive evaluation using PET nor an invasive assessment of coronary flow reserve (CFR) was carried out. This was because the ejection fraction was preserved, there were no abnormalities in resting or inducible wall motion, and the clinical picture was more in line with an anatomic coronary variant than microvascular dysfunction. PET‐based CFR assessment can improve the diagnostic clarity of suspected ANOCA, but it is usually used in patients who have ongoing angina despite normal coronary angiography or when initial noninvasive tests are conflicting or inconclusive [13, 14].

As reported in a case report by Khurana et al. [15], a patient with congenital absence of the left circumflex artery presented with postero‐inferior wall myocardial infarction. In a case reported by Fugar et al. [16], a patient who presented with transient loss of consciousness following a mechanical fall was found to have an absence of the left circumflex artery with a hyperdominant right coronary artery. Congenital absence of the left circumflex artery was found in patients who presented with non‐exertional chest pain after emotional stress in a case report published in 2015 by Varela et al. [17]. In the above‐mentioned case reports, patients with congenital absence of the left circumflex artery presented with postero‐inferior wall myocardial infarction, transient loss of consciousness, and non‐exertional chest pain due to emotional stress. In contrast, our patient's presentation with years of chronic dyspnea and nonspecific chest heaviness without ischemia has not been described previously and represents a distinct clinical presentation of CALCx.

## 4 Conclusion

Congenital absence of the left circumflex artery (CALCx) is a rare coronary anomaly that often presents with few or no symptoms, but can also manifest through a wide range of clinical features, such as angina‐like symptoms or chronic shortness of breath, as observed in the present case. This condition is frequently misdiagnosed as a complete atherosclerotic occlusion of the left circumflex artery. As such, advanced diagnostic techniques like coronary CT angiography and left heart catheterization are essential for accurate diagnosis and to avoid potential misinterpretation during coronary interventions.

This case is distinct from previously published cases because the patient presented with chronic dyspnea without ischemia, highlighting a lesser‐known clinical phenotype of CALCx. Moreover, the absence of standardized guidelines for diagnosing and managing CALCx highlights the need for personalized treatment strategies, as demonstrated in this case. To address these challenges, further research is required to establish comprehensive clinical guidelines, ultimately improving the care and outcomes for affected patients.

## Author Contributions

**Rohit Pandit:** conceptualization, data curation, formal analysis, investigation, supervision, validation, visualization, writing – original draft, writing – review and editing. **Ahmad Abu‐Haniyeh:** conceptualization, investigation, methodology, supervision. **Anil Nepali:** investigation, writing – original draft. **Nishchal Regmi:** visualization, writing – review and editing. **Heena Maharjan:** conceptualization, writing – original draft. **Ahmad El‐Moussa:** data curation, writing – review and editing.

## Funding

The authors have nothing to report.

## Ethics Statement

Ethical approval was not required according to national and institutional guidelines for single‐patient case reports.

## Consent

Written informed consent was obtained directly from the patient for publication and accompanying images.

## Conflicts of Interest

The authors declare no conflicts of interest.

## Data Availability

The data supporting this article's findings are available from the corresponding author upon reasonable request.
